# Experimental enhancement the evaporator performance using pulsated refrigerant flow

**DOI:** 10.1038/s41598-026-63916-y

**Published:** 2026-07-30

**Authors:** Osama A. Gaheen, Mohamed A. Aziz, Ahmed M. Elsayed, M. A. Abdelrahman, Mohamed A. Khalifa, A. Abd ELhamid, Haitham Elshimy

**Affiliations:** 1 Mechanical Engineering Department, Institute of Aviation Engineering and Technology, Giza, Egypt; 2 Mechanical Engineering Department, Faculty of Engineering, Suez University, P.O.Box:43221, Suez, Egypt; 3 Mechanical Engineering Department, Faculty of Engineering, Fayoum University, Fayoum, 63514 Egypt; 4 Mechanical Engineering Department, Faculty of Engineering, Shoubra Faculty of Engineering, Benha University, Benha, 11629 Egypt; 5 Mechanical Engineering Department, Institute of Aviation Engineering and Technology, Giza, Egypt; 6 Department of Computer Science, Faculty of Computers and Information, Suez University, P.O.Box:43221, Suez, Egypt; 7 Department of Mechanical and Aerospace Engineering, United Arab Emirates University, Al Ain, United Arab Emirates

**Keywords:** Pulsating flow, Pulsatile refrigerant, Heat transfer in Evaporator, COP, Air-conditioning, Pulse generation, Heat exchanger., Energy science and technology, Engineering

## Abstract

This study investigates the experimental application of pulsating flow to significantly improve heat transfer rates within an air conditioning system’s evaporator. The core aim is to boost the evaporator’s performance and, consequently, the overall coefficient of performance (COP) of the air conditioning unit. Refrigerant pulsations are generated within the evaporator coil using an electrical pulse control circuit, with tested frequencies of 6, 9, and 12 Hz under variable ambient temperatures of 24 °C, 26 °C, and 28 °C. Experiments conducted on a split air conditioner revealed only a negligible increase in compressor power consumption. Notably, the pulsating technique dramatically reduced the operating time needed to reach a desired temperature, with the most significant impact observed at a frequency of 9 Hz. This modification is characterized by its simplicity and cost-effectiveness, making it easily adaptable to various cooling and air conditioning systems. The results demonstrate a 13.33% increase in the evaporator’s heat transfer rate, a 13.63% improvement in the system’s COP, a 23.3% reduction in operating time, and consistent power consumption. These findings collectively highlight the effectiveness and practicality of integrating pulsating flow for enhancing the evaporator performance of an air conditioning system.

## Introduction

Recent advancements in air conditioning (AC) systems focus on improving energy efficiency and reducing power consumption while maintaining thermal comfort. However, the widespread use of AC has significantly increased electricity demand, especially during summer, where it can account for up to 70% of residential energy consumption, leading to higher peak loads and increased energy costs.

To address these challenges, pulsating flow has been widely investigated as an effective method to enhance the evaporator heat transfer. Pulsations improve fluid mixing, increase wall wetting, and reduce boiling boundary layer thickness, resulting in enhanced thermal performance. Several studies have demonstrated these benefits across different systems. Ye et al.^1^ reviewed pulsating flow mechanisms and highlighted the need for further investigation of governing parameters. Benavides et al.^2^ developed theoretical models for pulsating heat exchangers, while Jun et al.^3^, and Gaheen et al.^4^ experimentally showed that flow rate, geometry, and pulsation frequency significantly affect heat transfer. Additional studies confirmed similar enhancements in cylinders and thermal systems under pulsating conditions^5,6^.

Pulsating refrigerant flow has also shown promising results in refrigeration and air-conditioning applications^7–14^. Furthermore, Roh et al.^10^ achieved improvements in heating capacity and COP in heat pump systems using pulsation techniques. Wang et al.^15^ experimentally investigated the effect of pulsation width on heat transfer and pressure drop in an air-to-refrigerant evaporator. The results showed that pulsating flow enhanced the overall heat transfer coefficient by about 27% and the refrigerant-side heat transfer coefficient by 123%, while the average pressure drop remained nearly unchanged compared with continuous flow. The heat transfer enhancement was strongly influenced by the refrigerant mass flux and pulsation period. While Yang et al.^16,17^ demonstrated that pulsation parameters strongly influence pressure drop and flow regimes in two-phase systems.

The application of pulsating flow in refrigeration systems has attracted considerable attention in recent years. Yang et al.^18^ developed a response surface methodology-based model for predicting the liquid–vapor two-phase heat transfer coefficient of R134a during pulsating flow boiling in an evaporator. In addition, Lewis et al.^19^ investigated the behavior of mixed refrigerants undergoing pulsating flow in micro-coolers with pre-cooling and evaluated their thermal performance under such operating conditions. The effect of condenser cooling methods on pulsating heat pipe (PHP) performance has also been investigated by N. Iwata et al.^23^. The results indicated that natural convection provided lower thermal resistance than forced air convection, while both cooling methods exhibited similar performance at the same evaporator temperature Mahyar Fazli et al.^24^, presents a comprehensive review of PHP channel geometry and characteristics has also been presented. The study summarized recent experimental and numerical investigations and highlighted the significant influence of channel geometry on the thermal performance of pulsating heat pipes.

Pedram Vatankhah, Fatmeh Sari et al.^25^ show that periodic air injection can effectively enhance heat transfer in laminar channel flow when appropriate injection parameters are used. An optimum injection frequency and flow rate ratio improve thermal performance with limited friction losses, providing a promising approach for energy-efficient and controllable cooling applications. Oscillatory air injection significantly improved heat transfer, yielding enhancements of 62%, 220%, and 240% at Reynolds numbers of 200, 600, and 1000, respectively, compared with the no-injection case.

Indranil Brahma and Satbir Singh^26^ experimentally investigated pulsating heat transfer in a copper tube under various waveforms, frequencies, flow rates, and pressures. The results showed that pulsation can either enhance or reduce heat transfer compared with steady flow, depending mainly on the amplitude ratio and pulsating waveform. The heat transfer enhancement ratio ranged from 0.48 to 2.18, indicating that pulsation may significantly affect thermal performance. In addition, Long Short-Term Memory (LSTM) deep learning models were successfully employed to predict pulsating heat transfer and optimize waveform characteristics for improved performance.

Despite these promising findings, research on pulsating refrigerant flow in practical air conditioning systems remains limited, with most studies focusing on individual components rather than complete systems. Therefore, further investigation is needed to enable practical implementation and optimize system performance.

Most existing investigations have been limited to evaporators, capillary tubes, or laboratory-scale refrigeration systems, leaving a gap regarding the practical implementation of pulsating flow in commercially relevant air conditioning units. This study addresses this gap by applying pulsating refrigerant flow through evaporator modification to a 1.5 hp residential direct-expansion air-conditioning system with the aim of enhancing heat transfer and reducing compressor power consumption. ECO-22 was selected as the working refrigerant because of its lower environmental impact compared with conventional refrigerants such as R-22 and its compatibility with existing systems. The presented work represents a strong practical application of the proposed concept in a commercially relevant air-conditioning system, thereby providing new insight into its feasibility, energy-saving potential, and contribution to the development of more efficient and sustainable air-conditioning technologies under actual operating conditions.

## Description of the proposed work

The proposed technique addresses key limitations in current air conditioning systems, particularly the restricted heat transfer performance of air refrigerant evaporators, which reduces the overall Coefficient of Performance (COP) and increases energy consumption. Although pulsating flow has demonstrated superior heat transfer compared to continuous flow, its application remains limited, especially regarding the optimization of control parameters such as frequency. Additionally, challenges related to pressure fluctuations and flow interruptions in evaporators require further investigation.

This study introduces a novel approach by converting the refrigerant flow of ECO-22 from continuous to pulsating flow to enhance the indoor heat transfer rate. Pulsation is generated using a set of electric solenoid valves installed at the evaporator inlets, controlled to operate at frequencies of 6, 9, and 12 Hz. Based on the findings reported in previous studies^4–6,27,28^, pulsating refrigerant flow within this frequency range has demonstrated a positive effect on heat transfer and overall system performance. Therefore, the frequencies of 6, 9, and 12 Hz were selected in the present study because they represent a practically achievable operating range. The experimental work was conducted on a 1.5 Hp split air conditioner. A summary of previous studies on pulsating flow is provided in Table 1.

The properties of ECO-22 and the justification for selecting it as the working refrigerant are presented. ECO-22 was selected owing to its excellent thermodynamic performance, superior energy efficiency, lower operating pressures, reduced refrigerant charge requirement, and environmentally friendly characteristics, making it a promising alternative to conventional refrigerants such as R22 and R404A.

**Table 1:** Previous research on refrigerant pulsating flow.

Ref. No.	Application	Study type	Fluid	Pulsate frequency (Hz)	Pulse Mechanism	Enhancement	Limitations
Roh et al.^10^	Heat pump	Exp.	R-410 A	0.005	Control valve	Heating capacity increased by 4%; COP increased by 3.2%	• Variable frequency • analysis of local heat transfer • mechanisms • flow behavior
Bohdal et al.^20^	Heat exchanger	Exp.	R-404 A	0.029–0.07	Periodic-type disturbances	Reduced the active surface of heat transfer	• High frequency range • specific evaporator configuration
Chen et al.^21^	Flow boiling in a narrow annular duct	Exp.	R-134a	0.008:0.05	Intermittent flow boiling	Little effect on the time-averaged heat transfer coefficient	• High frequency range
Wang et al.^15,22^	Heat exchanger	Exp.	R-134a	0.016:10	Solenoid valve	Refrigerant-side heat transfer coefficients improved by about 123%	• flow visualization • operating conditions
N Iwata1 et al. ^23^	Pulsating Heat Pipe	Exp.	R -35	Not measured	self-excited oscillation of the fluid	natural convection	• Variable frequency • Multi capillary loops
Pedram Vatankhah Fatmeh Sari et al.^25^	periodic chanal flow	parametric study	air	1/900 :1/0.09	Pulsation was produced by oscillatory air injection	heat transfer was enhanced by **4–83%**	• Variable humidity levels • Variable cooling loads
Indranil Brahma^26^	turbulent pulsating pipe flow	Exp.	air	45	Reciprocation compressor	0.48 to 2.18 of steady flow, and maximum enhancement of 118%.	• Variable frequency • Variable Coolant fluid
Current Study	Air conditioning	Exp.	ECO-22	0:12	Solenoid valve	• Heating capacity increased by 13.33%; • COP increased by 13.63%. • Operating time reduced by 23.3%	• Variable humidity levels • Variable cooling loads • flow visualization

Table 2 compares the proposed pulsating flow approach with other evaporator enhancement techniques reported in the literature. Unlike conventional methods that require geometric modifications and may increase pressure losses, the present method enhances evaporator performance and COP through periodic flow modulation without altering the evaporator structure.

**Table 2:** Comparison of different evaporator enhancement techniques and the proposed pulsating flow approach.

Enhancement Technique	Enhancement Mechanism	Advantages	Limitations	Effect on Pressure Drop
Finned evaporator	Increased heat transfer area	High heat transfer enhancement; widely used	Increased size and manufacturing complexity	Moderate
Twisted tape inserts	Swirl generation and flow mixing	Improved convective heat transfer	Significant pressure loss; insertion required	High
Micro-fin tubes	Surface roughness and secondary flow generation	Enhanced boiling and heat transfer performance	Higher fabrication cost	Moderate
Nanofluids	Improved thermal conductivity of working fluid	Increased heat transfer coefficient	Stability and compatibility issues	Low–Moderate
Pulsating heat pipes	Oscillatory fluid motion	High thermal performance	Complex design and control	Low
Present study (Pulsating flow)	Periodic modulation of refrigerant flow rate	Improved evaporator performance and COP without structural changes	Requires flow control mechanism and frequency optimization	Low

## System design and experimental work

### Experimental configuration

The evaporator in the direct expansion (DX) air conditioning system operates as a heat exchanger with ECO-22 refrigerant and air as unmixed fluids. To apply pulsating flow, the evaporator coil was divided into two equal sub-coils, each equipped with a solenoid valve at its inlet. A custom electrical control circuit using timers and relays was designed to generate controlled pulses at different frequencies, enabling periodic opening and closing of the valves^27,28^.

**Fig. 1:**
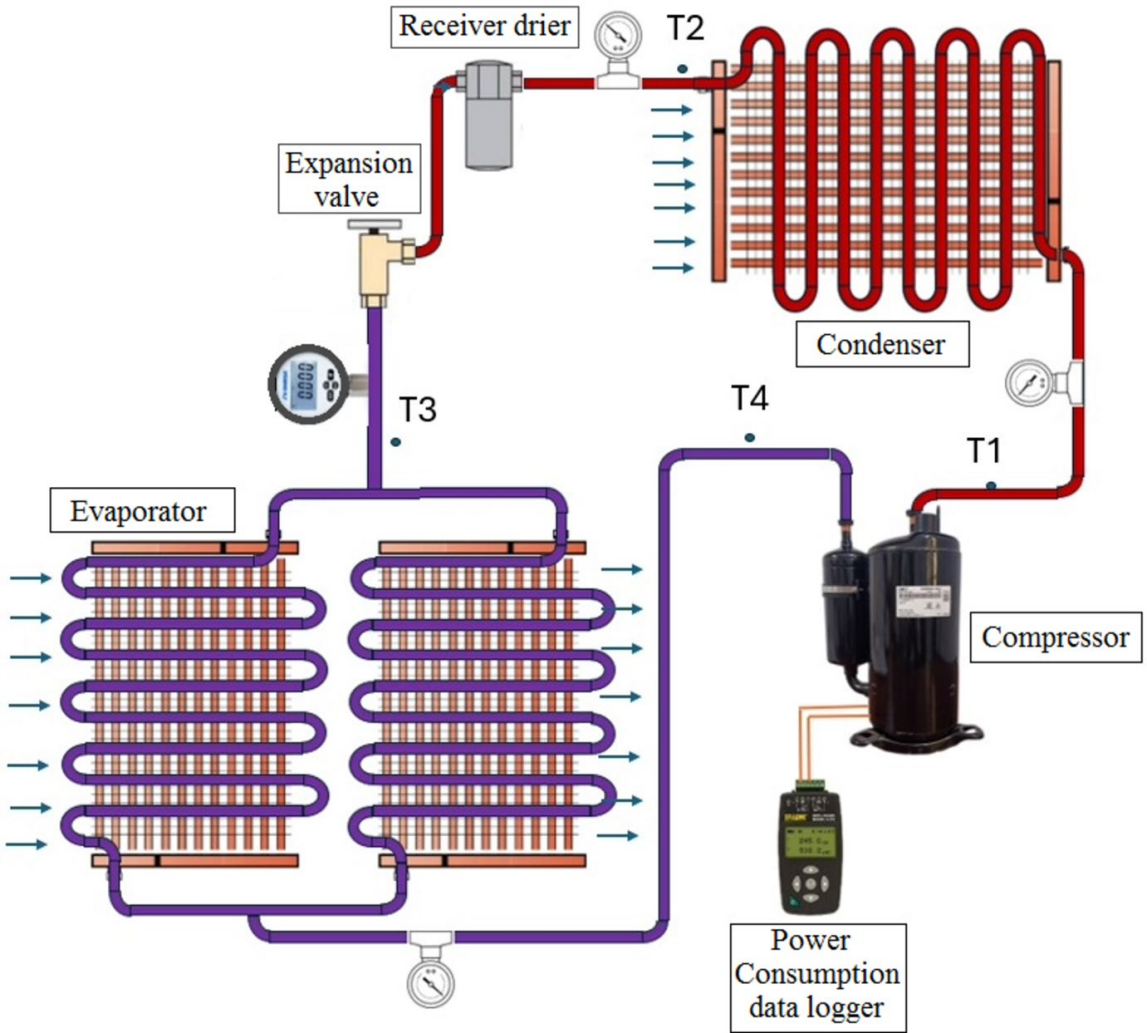
Schematic diagram for standard air conditioning cycle setup and main components.

This alternating operation for the two solenoids produces pulsating ECO-22 flow, which helps reduce pressure rise before entering the evaporator and modifies the heat transfer behavior and its modes. While the heat exchanger area and fluid properties remain constant, the pulsation is expected to enhance the overall heat transfer coefficient and cycle performance. The conventional AC system consists of a compressor, condenser, expansion device, and evaporator. In the present setup, the evaporator includes two parallel sub-coils supplied by a main feeder pipe, as shown in Fig. 1.

**Fig. 2:**
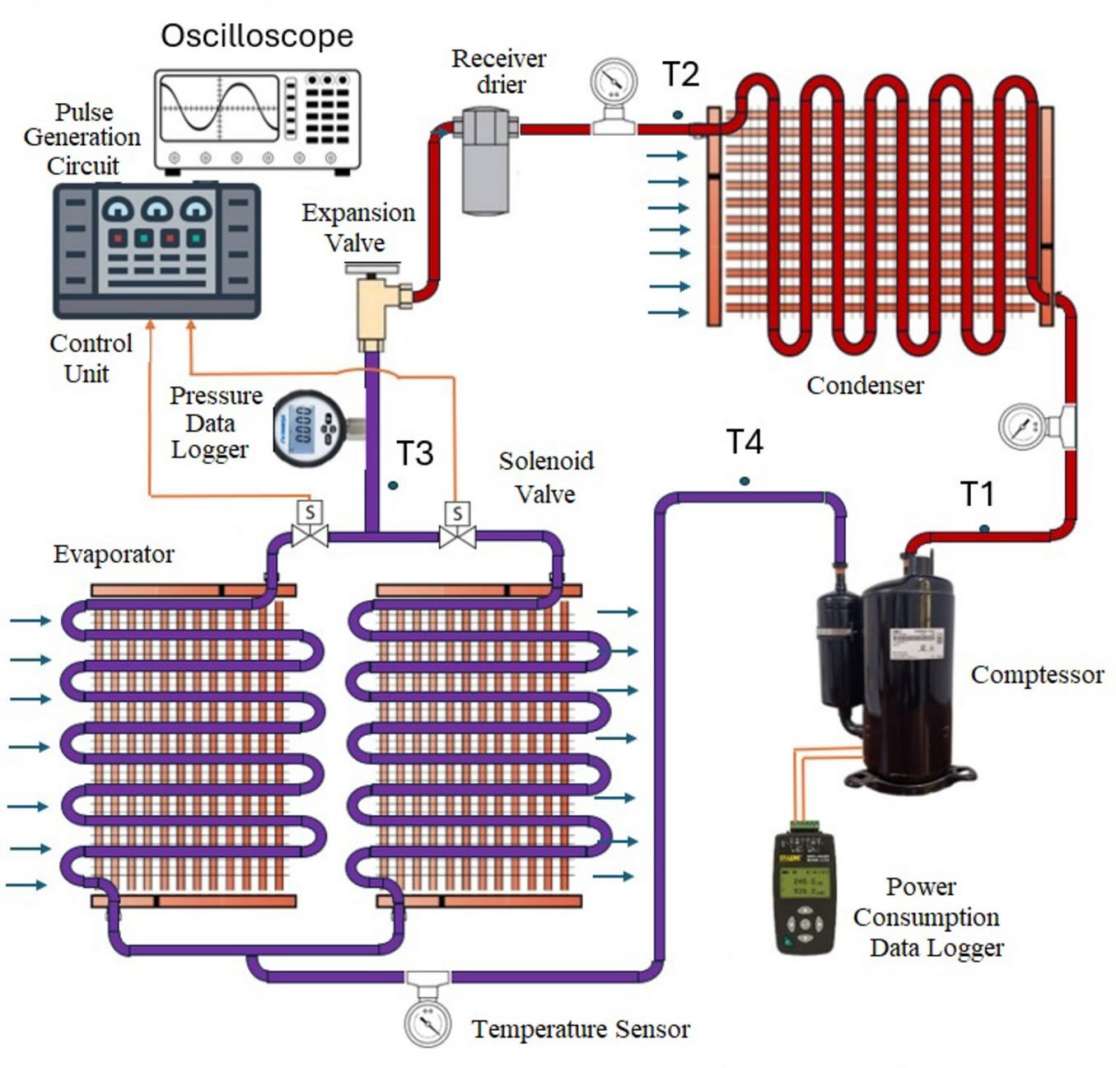
Schematic diagram of modified air conditioning cycle with pulsate mechanism.

**Fig. 3:**
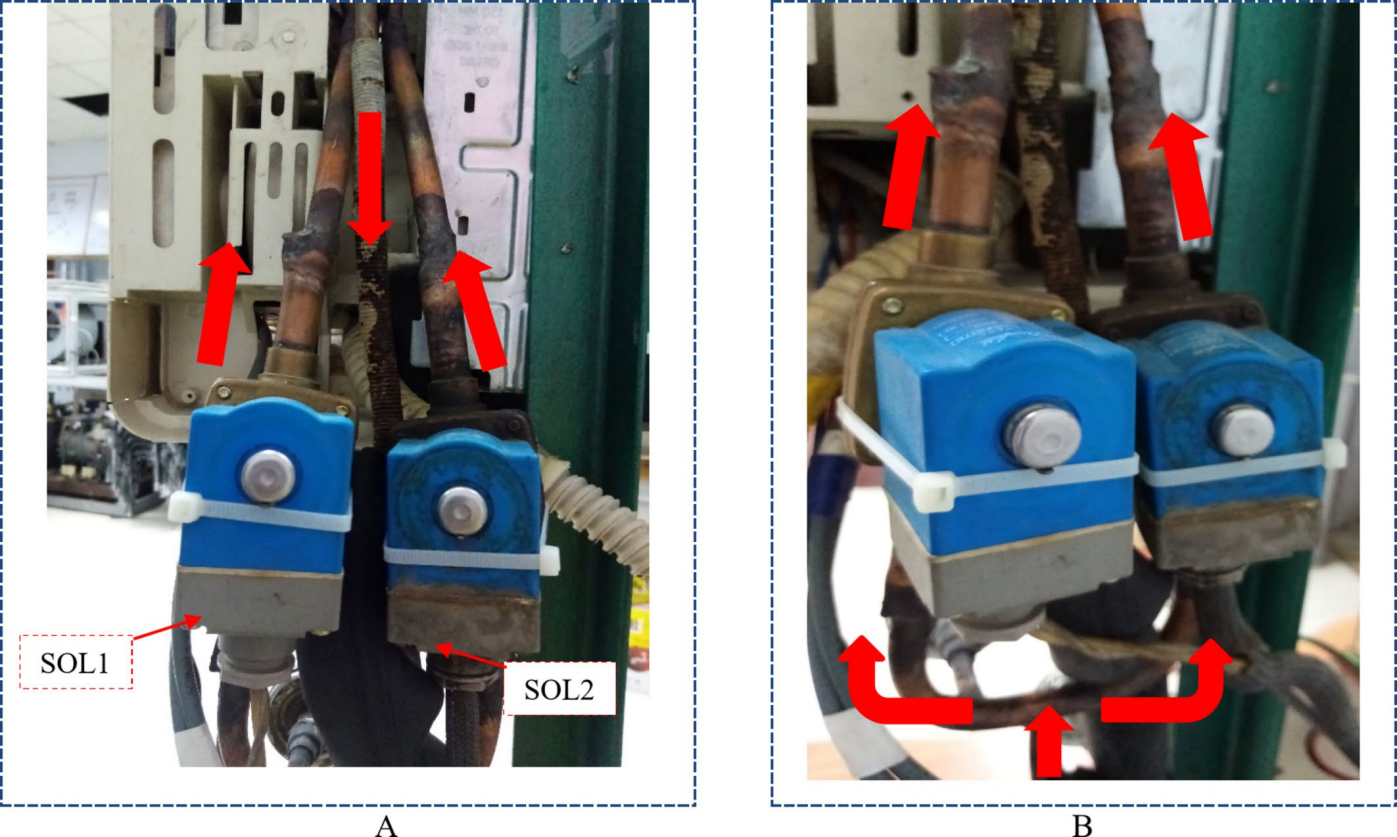
Inlet modification for main evaporator coil in DX split unit **A** Evaporator coil inlet and outlet refrigerant pipes **B** Solenoids inlet and outlets refrigerant pipes.

The primary modification to this standard cycle involves adding the two aforementioned solenoids at the inlet of the main evaporator coil. This main evaporator coil is thus divided into two equal sections. Each of these two sections has a separate inlet equipped with an electric solenoid, as shown in Figs. 2 and 3. Specifically, the main refrigerant line exiting the expansion device is split into two paths, each leading to one of the solenoids before entering the evaporator, as detailed in Fig. 3.

### Experimental apparatus and measurement procedure

Table 3 presents the specifications of the experimental apparatus and measuring instruments used in the present study, including the split air-conditioning unit, solenoid valve characteristics, data acquisition devices, and sensor accuracies. These details are provided to ensure the reproducibility and reliability of the experimental results.

**Table 3:** Specifications of the Experimental Apparatus Components.

No	Unit	Parameter	Specification
**1**	**Split Air Conditioner**	**Capacity**	1.5 HP
		**Cooling Capacity**	12,000 BTU/h
		**Coverage Area**	Up to 12–15 m²
		**Refrigerant**	ECO-22
		**Fan Speeds**	3-speed automatic control
		**Remote Control**	LCD wireless remote
		**Indoor Unit Dimensions**	Approximately 824 × 292 × 180 mm
		**Outdoor Unit Dimensions**	Approximately 677 × 531 × 282 mm
**2**	**Indoor unit**	**Cooling Capacity**	12,000 BTU/h (≈ 3.52 kW)
		**Refrigerant**	ECO-22
		**Tube Material**	Copper
		**Fin Material**	Aluminum with hydrophilic coating
		**Air Flow Rate**	Approximately 500–650 m³/h
		**Fan Speed Levels**	3-speed automatic control
		**Indoor Unit Dimensions**	824 × 292 × 180 mm (W × H × D)
		**Evaporator Surface**	Hydrophilic anti-corrosion fins
		**Expansion Device**	Capillary tube
		**Cooling Medium**	Forced convection air circulation
**3**	**The evaporator coil**	**Cooling Capacity**	12,000 BTU/h (3.52 kW)
		**Coil Type**	Finned-tube evaporator coil
		**Tube Material**	Copper
		**Fin Material**	Hydrophilic aluminum
		**Tube Outer Diameter**	7–9.52 mm (1/4–3/8 in)
		**Number of Rows**	2–3 rows
		**Fin Density**	12–16 fins per inch (FPI)
		**Tube Arrangement**	Staggered configuration
		**Expansion Device**	Capillary tube
**4**	**Electromagnetic solenoid valve coil**	**Voltage**	220–240 V AC, 50 Hz
		**Power Consumption**	8–20 W & 0.0545 Ah
		**Coil Duty**	Continuous duty (100%)
		**Insulation Class**	Class H
		**Protection**	Moisture-resistant encapsulated coil
		**Pressure drop**	20–35 kPa
		**Working Fluid**	Refrigerants such as R22, R410A, or ECO-22
		**Body Material**	Brass or copper valve body
		**Coil Color**	Blue

### Implementing refrigerant pulsation

Flow pulsation, or fluid oscillation, significantly influences heat transfer by reducing the sub-boiling layer in boundary layer thickness and thermal resistance, thereby enhancing performance^29,30^. Although many studies have investigated pulsating flow, most have focused on single-phase fluids such as water, oil, or air, with inconsistent conclusions regarding its effectiveness^31,32^. Notably, research on two-phase refrigerant pulsating flow remains limited. This study addresses this gap by applying controlled pulsating flow of refrigerant using a solenoid-valve system integrated into a real DX air conditioning unit.

### Experimental procedure and setup

**Fig. 4:**
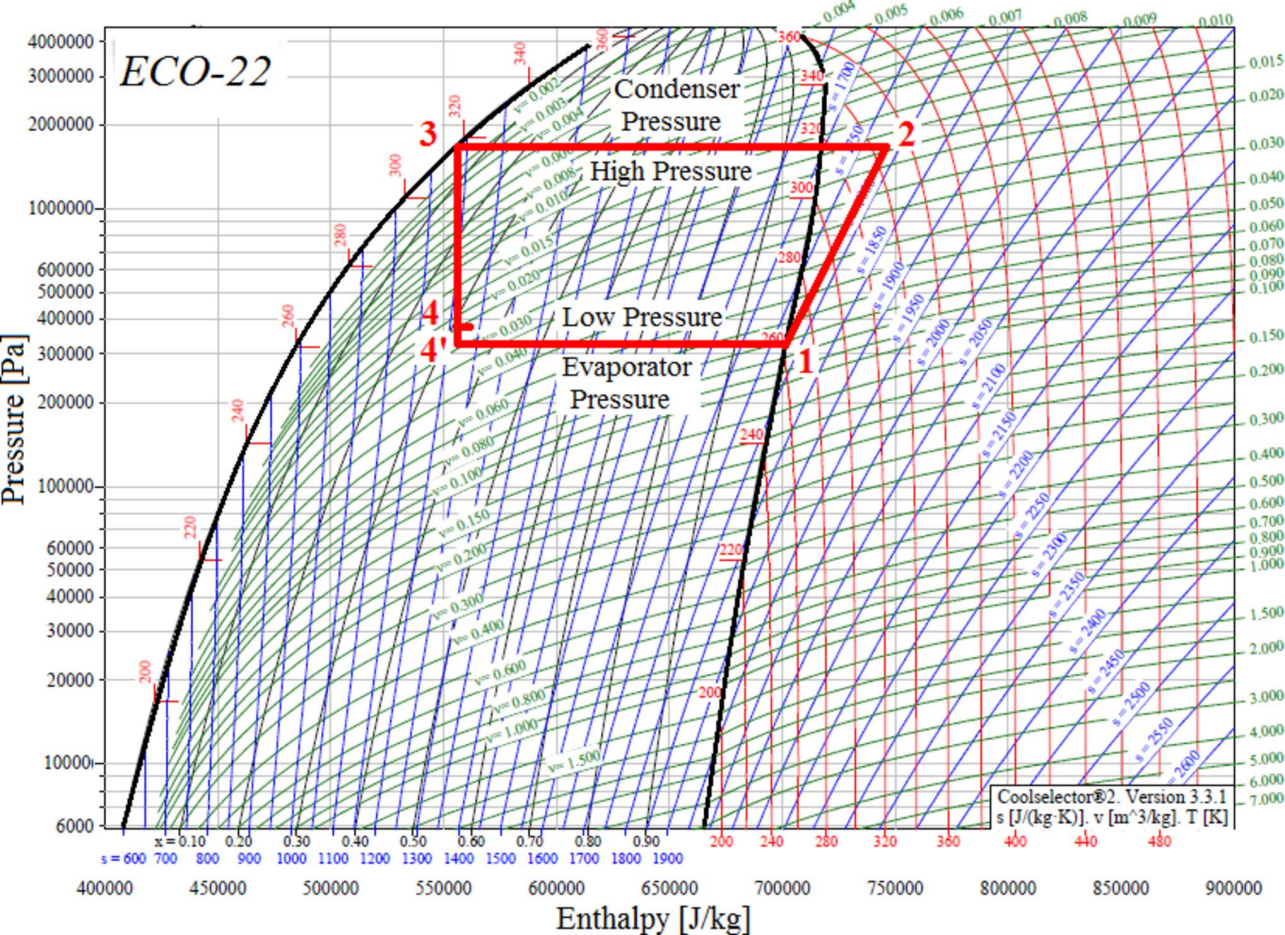
Schematic digram of actual air conditioning cycle on P-h diagram.

All experiments were conducted at the Heat Transfer Laboratory, Institute of Aviation Engineering and Technology, Giza, Egypt. The system setup (Fig. 4) applies pulsating refrigerant flow in a 1.5 HP split air conditioner using a simple classic control system with solenoid valves and a custom electrical circuit, enabling precise pulse control.

**Fig. 5:**
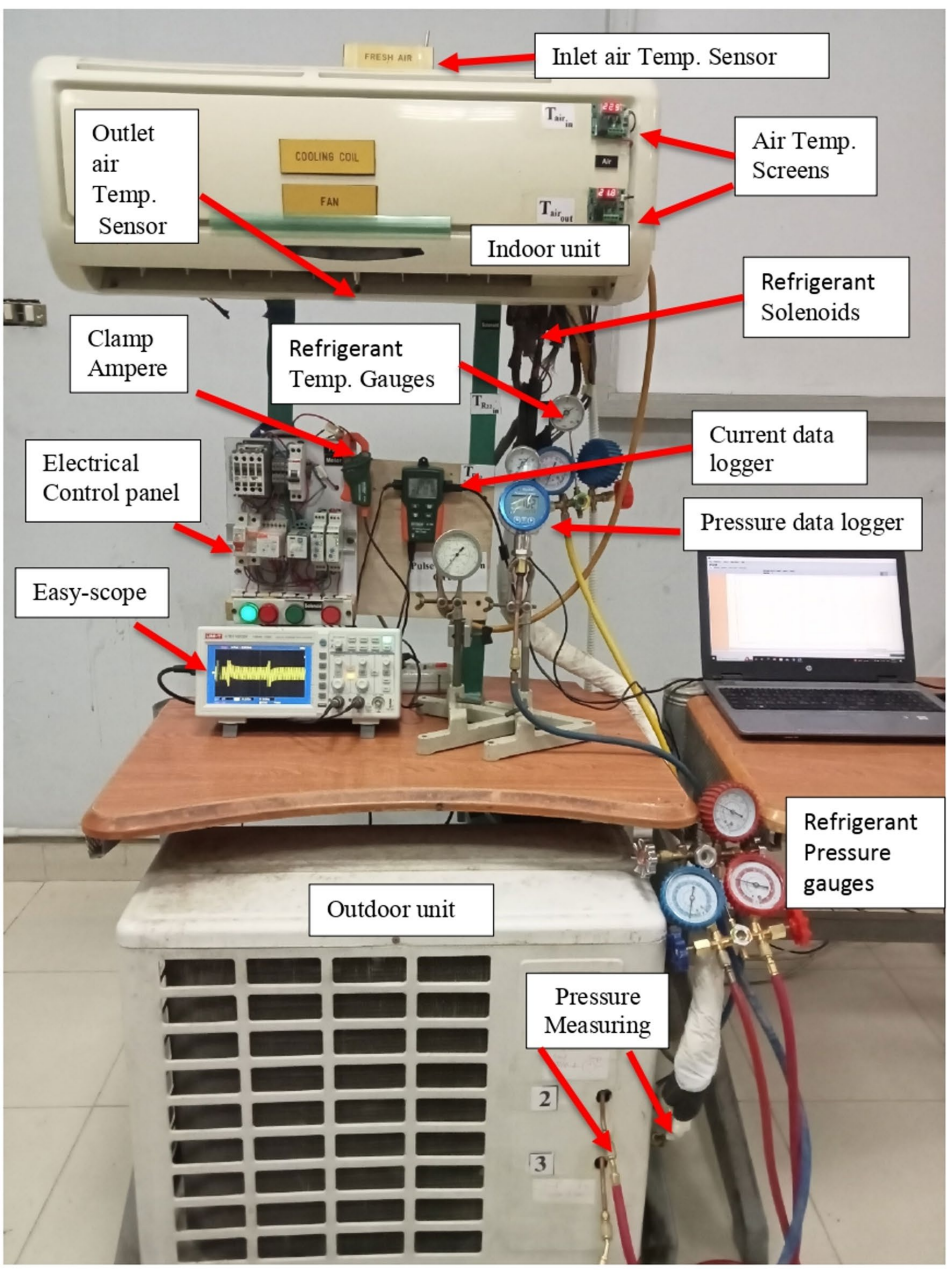
Actual setup and the experimental test rig.

Experimental results showed reduced power consumption, shorter operating time, and enhanced heat transfer in the evaporator, while maintaining stable pressure and phase behavior. Consequently, the Coefficient of Performance (COP) improved without increasing power consumption. Table 4 presents the measurement locations within the refrigeration cycle, corresponding to the P–h diagram in Fig. 4, which represented the experimental setup of Fig. 5.

**Table 4:** states the names and locations.

Port	Location
1	Evaporator exit point and compressor inlet
2	Compressor exit point and condenser inlet
3	Condenser exit point and expansion valve inlet
4	Expansion valve exit point and solenoid inlet

Table 5 summarizes the acoustic characteristics and long-term reliability of the system. The measured noise level ranged from 41 to 46 dB(A) under steady operation and increased slightly to 44–49 dB(A) during pulsating operation at 9 Hz. The corresponding increase of approximately 3 dB(A) indicates that the pulsation mechanism had a negligible impact on acoustic comfort. Continuous operation for approximately 1000 h, corresponding to more than 32 million switching cycles, revealed no refrigerant leakage, valve malfunction, or appreciable performance degradation, demonstrating the robustness of the pulsating-flow configuration.

**Table 5:** Comparison of acoustic characteristics for steady and pulsating flow operation.

Parameter	Steady Flow	Pulsating Flow (9 Hz)
Sound level, dB(A)	41–46	44–49
Test duration, h	1000	1000
Switching cycles	—	32.4 × 10⁶
Refrigerant leakage	None	None
Valve malfunction	None	None
Performance degradation	Negligible	Negligible

### Measuring instrumentations and specifications

**Fig. 6:**
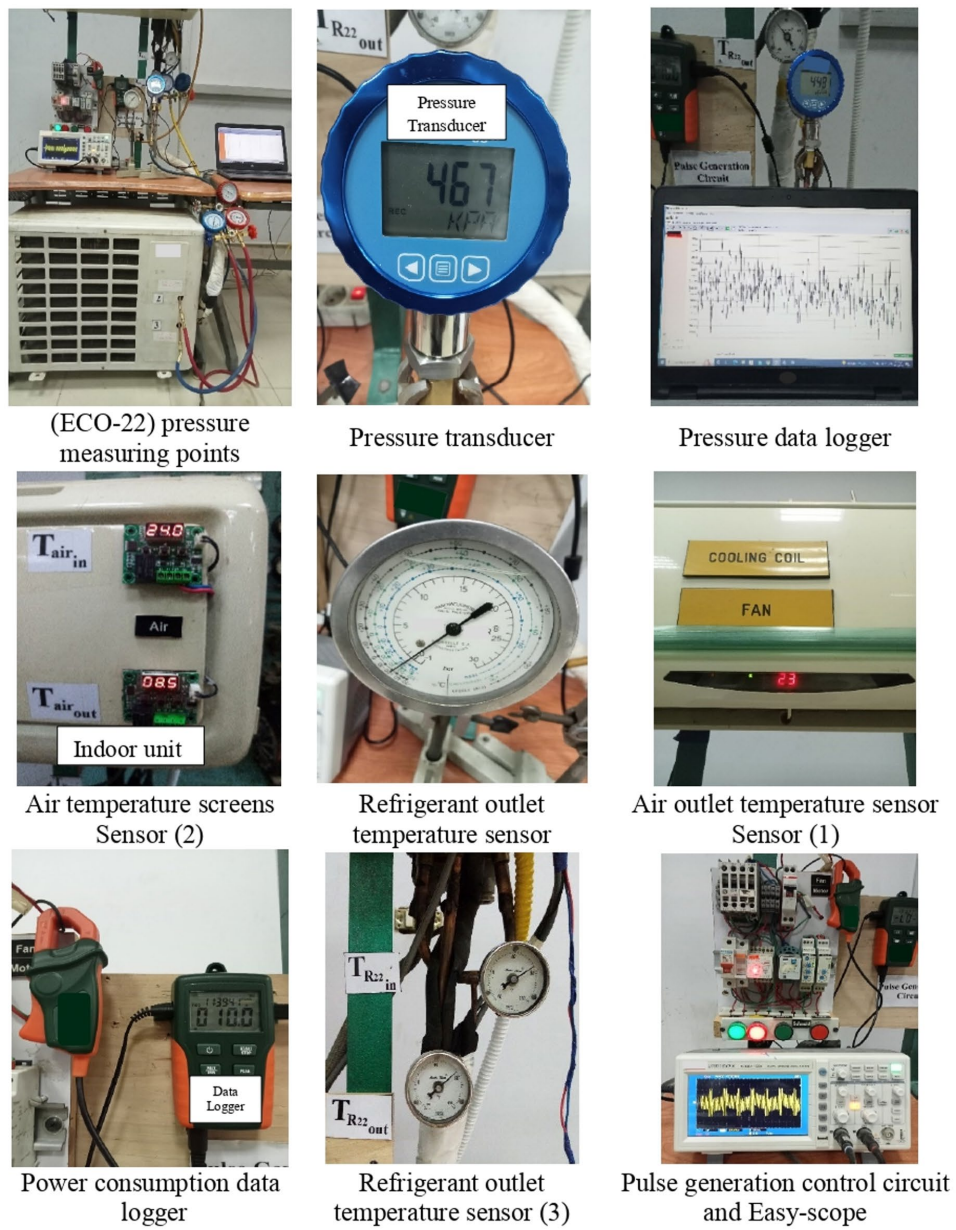
Experimental apparatus components.

Temperature and pressure were measured at all cycle states and ports using pressure and temperature data loggers. Air temperature was monitored at the inlet and outlets using multiple sensors for accuracy. Compressor power consumption was recorded using a current/voltage data logger. A custom electrical control circuit was designed to generate pulsation frequencies from 0 to 12 Hz for operating the solenoid valves. An Easy-scope was used to measure the frequency. All instruments are shown in Fig. 6.

The pressure measurements were obtained using a Track-It Data Logger (Pressure 350 D), with a range of 0 − 2500 kPa, a resolution of 0.09 kPa, a sampling rate of 10 samples/s, and an accuracy of ± 0.25% of the reading, ensuring precise monitoring of pressure variations with time during the application of pulsating flow in the experiments.

**Fig. 7:**
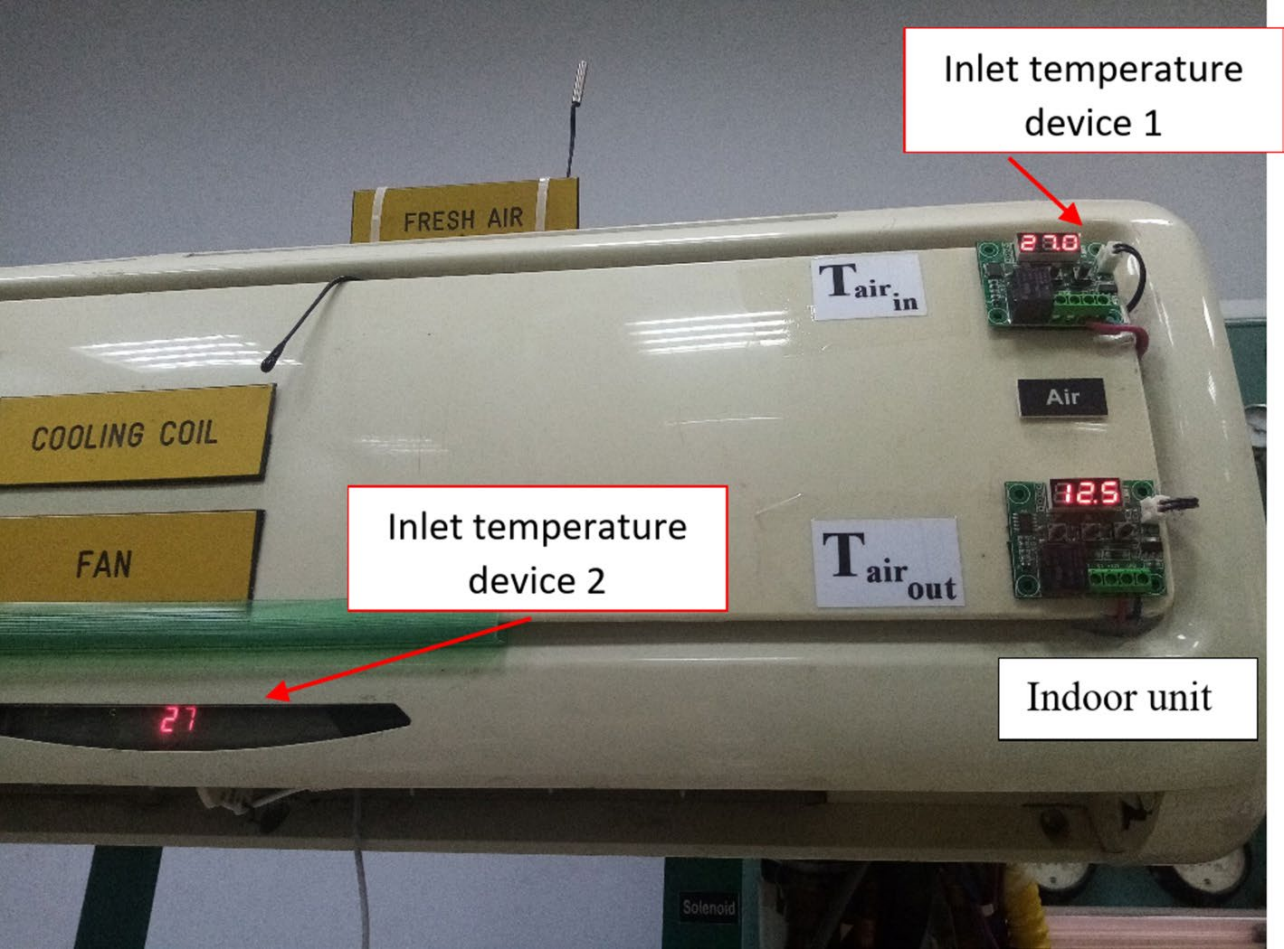
Multiple temperature measurement devices.

Multiple measurement platforms were used to ensure measurement accuracy through consistency of results. This is also considered a calibration method, employing different methods to measure the same object. This is evident in measuring temperatures before and after the evaporator using two different systems, as shown in Fig. 7.

### Electrical control circuit design for pulse generation

**Fig. 8:**
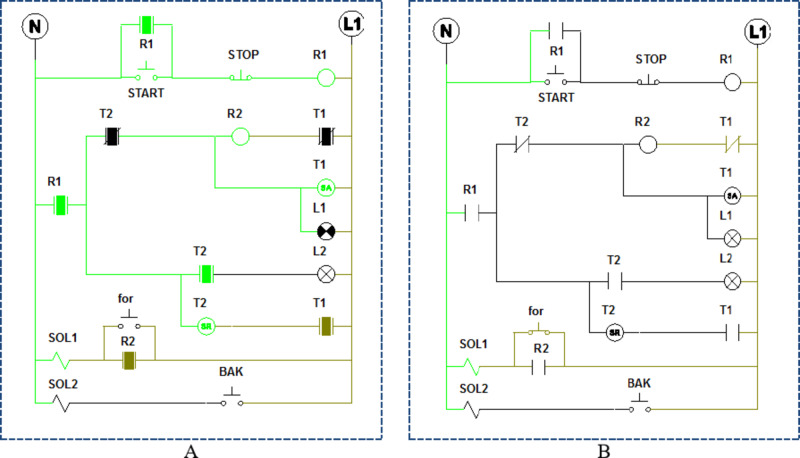
Schematic diagram of the electrical control circuit. **A** pulse generator activates & **B** pulse generator deactivates.

Pulsating flow was generated using a custom electrical control circuit composed of relays, timer on-delay, timer off-delay, contactors, and start/stop switches. High-speed 220 V AC two-solenoid valves, installed at the inlet of evaporator inlet, controlled the periodic opening and closing cycles. The circuit was designed to produce multiple frequencies in an alternating mode, as shown in Fig. 8. The Electric Control Techniques Simulator (EKTS) program was used to design and simulate the pulse signals, while an Easy Scope device was employed to measure and adjust the desired frequency.

### Uncertainty of the measurements

The precision of measurement tools significantly impacts the accuracy of experimental results, making error analysis and evaluation crucial. Coleman et al.^33^ introduced a standardized method for uncertainty analysis. When a result *K* depends on multiple parameters *x1*,*x2*,*.*,*xn*, the expanded uncertainty *H*_*K*_ can be mathematically expressed using Eq. (1):

1 $$\:{H}_{K}=\sqrt{{\left(\frac{\partial\:K}{\partial\:{x}_{1}}{H}_{{x}_{1}}\right)}^{2}+{\left(\frac{\partial\:K}{\partial\:{x}_{2}}{H}_{{x}_{2}}\right)}^{2}+\dots\:\dots\:..+{\left(\frac{\partial\:K}{\partial\:{x}_{n}}{H}_{{x}_{n}}\right)}^{2}}$$

During the continuous experimental procedures, data were collected for time, pressure, and temperatures of ECO-22 and air at various frequencies. Table 6 provides details regarding the precision of the measuring instruments and the corresponding uncertainty for both measured and computed parameters. Error analysis was performed for mass flow rate, Coefficient of Performance (COP), rejected heat rate from air, and electrical power consumption.

**Table 6:** Assessing the precision of measuring instruments and evaluating the uncertainty associated with both measured and computed experimental data.

Measured parameters	Instrument	Resolution	Range	Sample rate	Accuracy	Measured Accuracy
I (Amper) V (AC Volt)	Data logger device measuring current and volt	0.1 A 0.1 V	1 : 200 A 10 : 600 V- DC	1 Sample/ s	± 1 A ± 1.5 V	0.20 0.25
Pressure	Track-It data logger (Pressure 350 D)	0.09 kPa	0:2500 kPa	10 Sample/s	± 0.25% reading	0.5
Air velocity	Pitot tube Anemometer/Defferential	0.01 m/s	1:80 m/s	2 Sample/s	± 2.5%rdg	0.2
Temperature	DC 12 V LED Digital Temperature Sensor	0.1 °C	-50 : 110 °C	0.5 s	± 0.1 °C	0.1
Frequency	Digital storage Osciliscope	0.1 Hz	2ns/div~50s/div 100 MHz	1GS/s	1mV/div~20 V/div	0.0
Time	Stopwatch	0.01 s	Open range	-	± 0.1 s	0.2
Length	Measuring tape	1 mm	0:20000 mm	-	± 0.1 mm	1 mm
**Computed parameters**	**Unites**	**Uncertainty**				
$\:{\dot{m}}_{air}$	kg/s	0.4%				
Q	W	0.7%				
COP	-	0.9%				
Compressor Power	$\:{W}_{comp}$	0.3%				
Cooling capacity	$\:{q}_{air.}$	0.7%				

## Mechanism of heat transfer and data processing

The specific enthalpy of the air conditioning cycle is determined from the measured pressures and temperatures at the evaporator and condenser inlet and outlet. A schematic representation of the cycle on the P-h chart is shown in Fig. 5. The estimated specific enthalpies are used to calculate the following parameters: The sensible heat rejected from the air ($\:{Q}_{rej\left(air\right)}$) is calculated by:

2 $$\:{Q}_{rej\left(air\right)}+{L.H}_{air}={Q}_{added\left(ECO-22\right)}$$

where the latent heat of air $\:\left({L.H}_{air}\right)\:$is neglected, and $\:{Q}_{added\left(ECO-22\right)}$ represents the heat add to the refrigerant.

3 $$\:{\dot{m}}_{ECO-22}{(h}_{1}-{h}_{{4}^{{\prime\:}}})={\dot{m}}_{a}{Cp}_{a}\left({T}_{{a}_{in}}-{T}_{{a}_{out}}\right)$$

4 $$\:{\dot{m}}_{a}={\rho\:}_{a}{V}_{a}{A}_{air\:out\:}$$

$\:{\dot{m}}_{a}\:,\:{\rho\:}_{a}\:,\:{V}_{a\:,\:and\:}{A}_{a}$ represent the mass flowrate, density, and velocity of the air. On the other hand, $\:{A}_{air\:out\:}$is the air outlet area for the indoor unit.

The sensible heat added to the refrigerant, $\:{Q}_{added\left(ECO-22\right)}$. This heat added is less than the latent heat of vaporization of the refrigerant.

$$\:{Q}_{added\left(ECO-22\right)}<latent\:heat\:\left({\dot{m}}_{ECO-22}{h}_{fg}\right)$$

5 $$\:{Q}_{evaporator}={\dot{m}}_{ECO-22}({h}_{1}-{h}_{4{\prime\:}})$$

6 $$\:{W}_{compressor}={\dot{m}}_{ECO-22}({h}_{2}-{h}_{1})$$

7 $$\:COP=\frac{{Q}_{evaporator}}{{W}_{compressor}}$$

8 $$\:COP=\frac{{h}_{1}-{h}_{4{\prime\:}}}{{h}_{2}-{h}_{1}}$$

Where ($\:{h}_{1}-{h}_{{4}^{{\prime\:}}})\:$represents a portion of latent heat. The mass flow rate of air was calculated by measuring the velocity using a Pitot tube and measuring the exit area for air from the evaporator outlet. It is observed that at an air velocity of 4.5 m/s and an exit area of 360 cm², the mass flow rate of air was 198 g/s, considering the air density at a constant temperature of 26 °C and atmospheric pressure at sea level. The compressor power is calculated by measuring the consumed current and measured voltage. A power factor of cos θ = 0.95 for the public network is used.

9 $$\:{W}_{comp}=IV\mathrm{c}\mathrm{o}\mathrm{s}\theta\:$$

## Validation of the pulsation applicability

A related previous study^7^ investigated the enhancement of heat transfer in a tube-in-tube heat exchanger using pulsating flow, conducting experiments under parallel, counter, and pulsating counter flow configurations. Significant findings included substantial improvements in heat transfer coefficients when transitioning from parallel to counter flow, with further enhancements observed with pulsating counter flow. Pulsating flow proved superior, yielding a 75% increase in the overall heat transfer coefficient, a 13.8% rise in sensible water cooling capacity, and a 13.4% improvement in the COP. It also demonstrated a reduction in pressure drops compared to continuous flow, indicating energy savings and improved fluid flow efficiency. The Darcy friction factor decreased by 5.3% at an optimal frequency of 9 Hz, balancing reduced frictional resistance with energy efficiency. Furthermore, pulsating intervals enhanced fluid-wall interactions, further improving heat transfer rates. These findings collectively underscore the importance of frequency optimization for maximizing heat transfer enhancement while minimizing energy consumption.

## Results and discussion

### Influence of pulsation frequency on refrigerant pressure

**Fig. 9:**
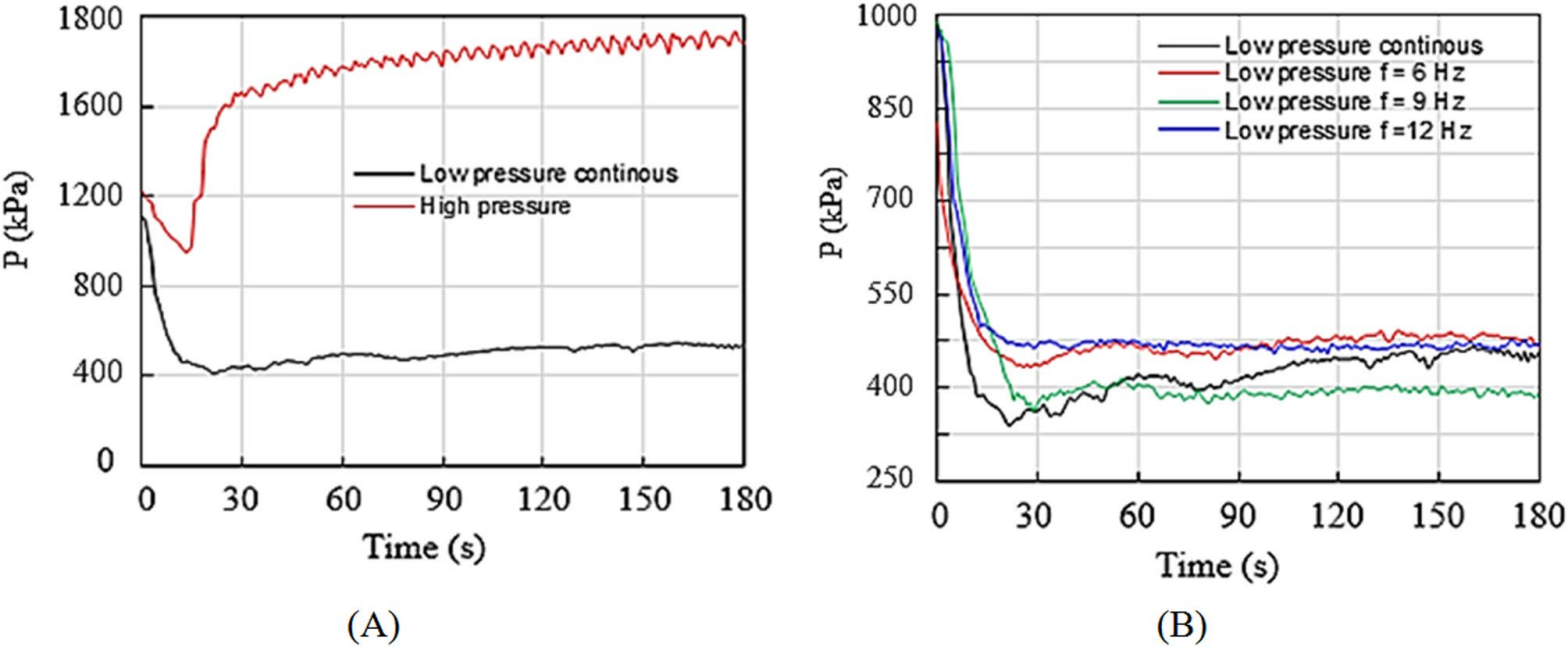
Effect of solenoid frequency on the pressure after and before the evaporator coil: **A** continuous high and low pressure, and **B** continuous, 6, 9, and 12 Hz low pressure (kPa).

The pressure both upstream and downstream of the evaporator coil was monitored using a pressure data logger to assess how the integration of solenoids, positioned before the evaporator inlet, impacts the Pressure-Enthalpy (P-h) diagram and, consequently, the Coefficient of Performance (COP) of the ECO-22 cycle. Figure 9 illustrates the observed pressure variations. P1 represents the low pressure at the evaporator exit and compressor inlet, while P3 denotes the high pressure at the expansion valve inlet. Sub-figures within Fig. 8 display different operational scenarios: (a) depicts continuous flow, and (b) shows the effects at frequencies of 6 Hz, 9 Hz, 12 Hz, and continuous flow, respectively. These visual representations offer crucial insights into the dynamic changes in pressure distribution, which are essential for understanding the system’s thermodynamic behavior and optimizing the COP.

The observed changes in pressure dynamics with varying solenoid frequencies reveal distinct patterns, providing valuable insights into the system’s response to pulse flow. The visual representation in Fig. 8 is a critical tool for understanding the nuanced relationship between solenoid frequency and pressure characteristics. This understanding is pivotal for fine-tuning and optimizing the overall performance of the air conditioning system, ensuring the pulse flow mechanism operates at an ideal frequency to enhance efficiency and achieve optimal outcomes in the P-h diagram. The total pressure drop (*ΔP*_*tot*_) is given by the sum of the evaporator pressure drop (*ΔP*_*evaporator*_) and the solenoid-measured pressure drop (*ΔP*_*solenoid*_), which equals 30 kPa, as shown in Eq. (10):

10 $${\it \Delta} P_{tot} = {\it \Delta} P_{evaporator} + {\it \Delta} P_{solenoid}$$

The cyclic opening and closing of the solenoid valve generated pulsating refrigerant flow within the evaporator, resulting in periodic fluctuations in pressure and mass flow rate. During the valve opening period, refrigerant entered the evaporator and the pressure increased, whereas valve closure temporarily restricted the flow and reduced the evaporator pressure. Consequently, the system operated under a periodic steady-state condition in which pressure and temperature oscillated around constant mean values. These pressure pulsations enhanced liquid-vapor mixing and disrupted the thermal boundary layer, thereby improving boiling heat transfer and increasing the evaporator heat absorption capacity. The enhanced refrigeration effect resulted in a higher coefficient of performance and reduced compressor power consumption compared with conventional steady flow operation.

Table 7 provides a comprehensive overview of the steady-state pressure values corresponding to different frequencies, comparing them with the normal continuous case. In the continuous case, the solenoid valves at the evaporator entrance remain continuously energized, maintaining specific pressure and temperature values at the evaporator’s inlet and outlet. Conversely, the solenoid’s operating frequency, alternating between energized and de-energized states, distinctly affects the pressure and temperature values at the evaporator’s inlet and outlet. Lower frequencies correlate with increased pressure and temperature before the evaporator, while higher frequencies result in a decrease until reaching values similar to the continuous case.

**Table 7:** Pressure steady-state values for different frequencies compared with normal continuous case.

Case	Continuous	6 Hz	9 Hz	12 Hz
High Pressure (kPa)	1792.61	1805.57	1810.77	1798.25
Low Pressure (kPa)	0443.33	0474.32	0410.92	0452.97

**Fig. 10:**
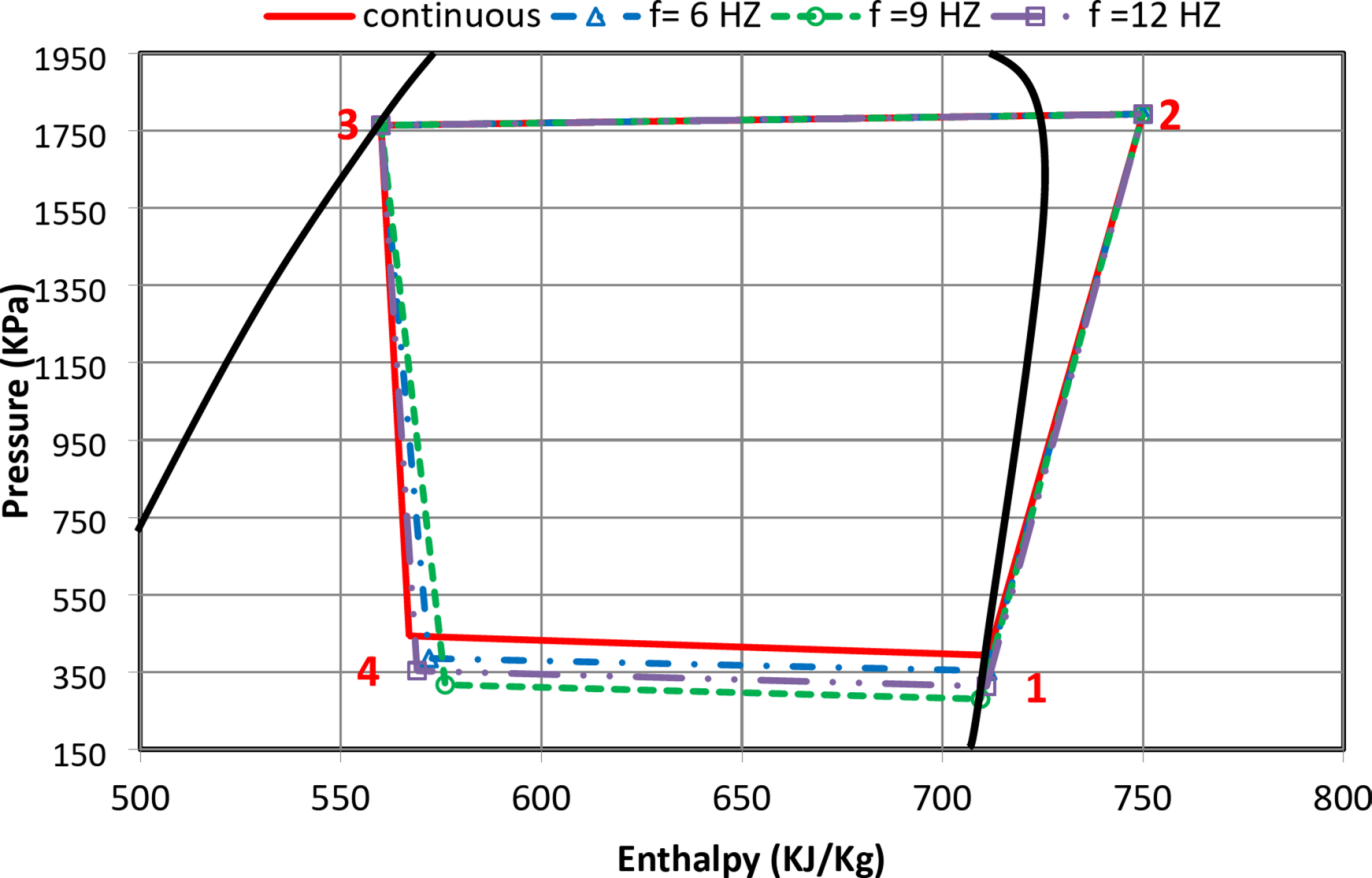
Comparison of the pressure–enthalpy (P–h) diagrams of the ECO-22 refrigeration cycle for continuous refrigerant flow and pulsating refrigerant flow at frequencies of 6, 9, and 12 Hz.

Figure 10 shows that the application of pulsating refrigerant flow enhances the refrigeration effect (h_1_-h_4_) while maintaining nearly constant compressor work (h_2_-h_1_). As a result, the coefficient of performance (COP) of the system increases compared with continuous flow operation. The highest COP is obtained at a pulsation frequency of 9 Hz, where the largest refrigeration effect is achieved, whereas 6 Hz and 12 Hz also provide higher COP values than the continuous-flow case. These results demonstrate that pulsating refrigerant flow improves the thermodynamic performance of the vapor-compression cycle, with 9 Hz representing the optimum operating frequency.

### impact on coefficient of performance (COP) and cooling capacity

**Fig. 11:**
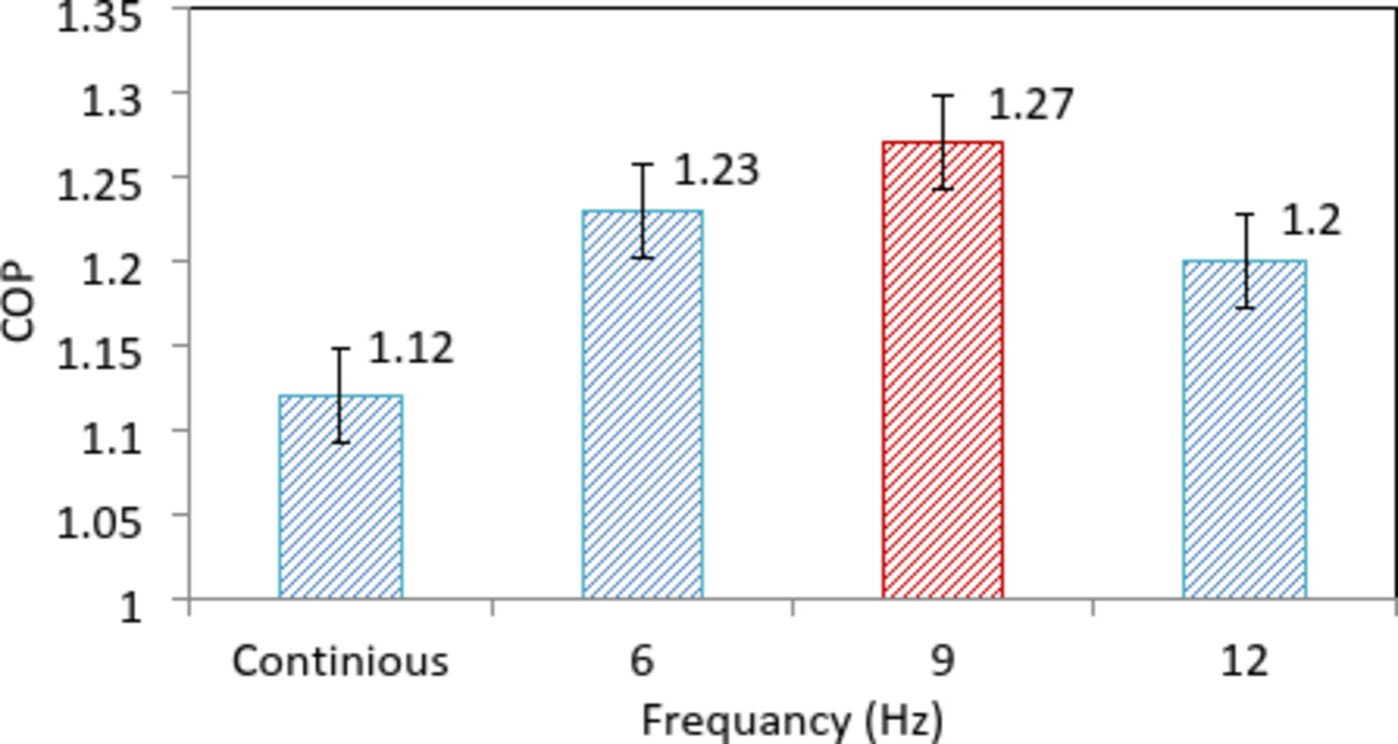
The COP values for the normal cycle and different frequencies.

For each operating frequency, three repeated measurements were performed for temperature, pressure, air flow rate, ambient temperature, power consumption, and the average values were used in the subsequent calculations. Figure 11 presents the Coefficient of Performance (COP) values for the normal cycle, 6 Hz, 9 Hz, and 12 Hz, which are 1.12, 1.23, 1.27, and 1.20, respectively, including the error bars. The COP increases with the application of pulsating ECO-22 refrigerant, reaching its maximum value at a frequency of 9 Hz. Beyond this point, a slight decline in COP is observed at 12 Hz, although it remains higher than the normal cycle. The overall percentage increase in COP is approximately 13.63%, demonstrating a clear improvement in system efficiency. These results indicate that an optimal pulsation frequency exists (around 9 Hz), at which the system achieves its best performance, rather than continuously increasing with higher frequencies.

Pulsating refrigerant flow enhances heat transfer in the evaporator by periodically disturbing the thermal boundary layer and promoting stronger mixing between the liquid and vapor phases. This improves refrigerant distribution, facilitates bubble detachment during boiling, and minimizes stagnant regions, thereby increasing the convective heat transfer coefficient and the refrigeration effect. As a result, the cooling capacity increases while the compressor power remains nearly unchanged, leading to higher COP and lower energy consumption compared with conventional steady flow. Among the investigated frequencies, 9 Hz provided the optimum performance because it established a favorable balance between flow disturbance and refrigerant residence time, maximizing boiling heat transfer and improving two-phase flow distribution.

However, increasing the pulsation frequency to 12 Hz resulted in a slight reduction in performance. At this frequency, the opening and closing cycles of the solenoid valve become very rapid, causing the flow behavior to approach that of continuous flow. Consequently, the periodic disturbances responsible for enhanced mixing and boundary-layer disruption become less effective. In addition, excessive flow oscillations and pressure fluctuations may increase hydraulic losses and reduce the effective time available for refrigerant evaporation. Therefore, the beneficial effects of pulsation are partially offset, resulting in a slight decrease in cooling capacity and COP compared with the optimum condition obtained at 9 Hz.

Table 8 compares outlet air temperatures under the same ambient conditions, including variations in air temperature, mass flow rate, specific heat, and cooling capacity. Results show that increasing refrigerant pulsation frequency reduces outlet air temperature and enhances cooling capacity, indicating improved system performance. Higher frequencies lead to greater cooling effectiveness, with a maximum cooling capacity improvement of 13.33% at 9 Hz compared to continuous flow, confirming the effectiveness of the pulsating flow technique.

**Table 8:** Comparison of the outlet air temperatures at ambient temperature of 26 °C.

Case	Continuous	9 Hz
**Inlet air temp (** ^**o**^ **C)**	26	26
**Outlet air temp (** ^**o**^ **C)**	13.5	11.7
**m**_**air**_ **(g/s)**	198	198
**Air speed (m/s)**	4.5 m/s	4.5 m/s
**Cp**_**air**_ **(J/kg.K)**	1005	1005
**ΔT (** ^**o**^ **C)**	12.5	14.3
**Q**_**rej air**_ **(W)**	2692.5	3051.5
**% increase in Q**_**rej**_	-	13.33
**COP**	1.12	1.27

### Power consumption and operating time analysis

**Fig. 12:**
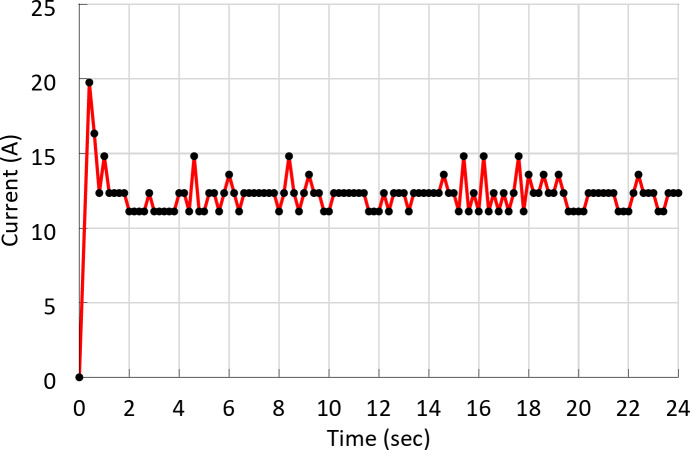
The consumed power recorded under pulsating flow conditions.

Power consumption varies with the flow type and is more fluctuating under pulsating conditions. It is measured experimentally using voltage/current data loggers or power analyzers, which provide real-time energy usage. The consumed power is calculated from measured voltage and current; for example, an average current of 12.3 A at 208 V corresponds to approximately 2440 W (Fig. 12), with minor variations due to factors such as power factor. The measured power is then used to evaluate the Coefficient of Performance (COP), defined as the ratio of useful cooling or heating to the input energy.

Table 9 provides a comprehensive comparison of consumed power and operating time across different cases: continuous flow and 9 Hz. The table outlines the peak current, air temperatures, temperature differences, time, time required for a one-degree Celsius decrease, target outlet air temperature, and operating time savings percentage for each case. Notably, at higher frequencies, there is a reduction in operating time, leading to energy savings and improved efficiency, with a significant 23.33% reduction in operating time at 9 Hz compared to the continuous case.

**Table 9:** Comparison between the consumed power and operating time at ambient temperature of 24 °C.

Case	Continuous	9 Hz
Maximum peak current (A)	10.3	10.3
Inlet air temp (^o^C)	24	24
ΔTmax (^o^C)	13.2	14
Time (min)	15	13
Time for one Celsius = Time (min)/ΔTmax (^o^C)	1.11	0.849
Target outlet air temp. (^o^C)	Outlet air temp (^o^C) Continuous case (13.5)	
Operating time to reach target outlet air temp. (min)	15	11.5
Operating time saving percentage %	0	23.3%

### Techno-economic analysis

The cost of adding the solenoid to generate the pulsed flow of the refrigerant can be calculated, and then the payback period can be calculated by comparing the cost of the addition with the annual savings resulting from reduced electricity consumption and improved performance factor (COP) of the air conditioner.

The total cost includes:

- Solenoid valve cost.
- Electrical control circuit cost (timer-on delay, timer-off delay, Relay).
- Installation and wiring costs.
- Running cost.

Table 10 presents a comparison of the annual operating cost between a conventional air conditioner and the proposed system with a 13.66% enhancement in COP. For the same cooling demand, the increase in COP resulted in a 12.02% reduction in electrical energy consumption, as calculated using Eq. (11). On the other hand, the initial cost of the solenoid and electrical control circuit can be negligible compared to running cost.

11 $$\:\mathrm{E}\mathrm{n}\mathrm{e}\mathrm{r}\mathrm{g}\mathrm{y}\:\mathrm{R}\mathrm{e}\mathrm{d}\mathrm{u}\mathrm{c}\mathrm{t}\mathrm{i}\mathrm{o}\mathrm{n}=1-\frac{1}{\mathrm{M}\mathrm{o}\mathrm{d}\mathrm{i}\mathrm{f}\mathrm{i}\mathrm{e}\mathrm{d}\:\mathrm{C}\mathrm{O}\mathrm{P}}$$

**Table 10:** Comparison of annual operating cost between a conventional 1.5 HP air conditioner and a 1.5 HP unit with a 13.66% COP enhancement.

Parameter	Conventional AC	AC with Pulsating Flow (Modified COP )
Cooling capacity	**1.5 HP**	**1.5 HP**
COP	1.12	1.27
Relative power consumption	100%	87.98%
Annual electricity consumption	E	0.8798E
Annual operating cost	C	0.8798 C
Annual cost saving	—	0.1202 C
Percentage reduction in annual running cost	—	12.02%

### Overall performance and future outlook

The current approach of pulsating flow in direct expansion air conditioning systems proves to be a novel and effective technique, demonstrating multiple positive impacts. The COP and heat transfer rate experience notable enhancements with an increase in pulsatile refrigerant frequency, contributing to improved overall system efficiency. Despite this enhancement, the increment in compressor power consumption remains negligible. Notably, the proposed modification significantly reduces the working time required to achieve a specific temperature, especially at higher pulsatile frequencies, leading to a substantial 23.3% decrease. The simplicity of the construction and the low-cost applicability of this technique make it adaptable to various cooling or air conditioning systems. Careful measurements at all points ensure operational safety, preventing pressure overloads beyond design limits for the compressor. In conclusion, the proposed modifications yield positive impacts on critical parameters, including a 13.33% increase in cooling capacity, a 13.63% improvement in COP, and a 23.3% reduction in operating time, while the consumed power remains constant, as presented in Table 11.

**Table 11:** Performance Parameters Enhancement.

Parameter	Modification effect	Percentage
Q_rej air_	Increased	13.33%
COP	Increased	13.63%
Operating time	Decreased	23.30%
Consumed power	Remains constant	00.00%

The proposed modifications exhibit minimal limitations from technical, commercial, and regulatory perspectives, indicating ease of implementation and broad applicability. A minor drawback is the mechanical noise generated by the solenoid valves; however, this is reduced during ECO-22 flow operation and can be further mitigated by proper installation near the outdoor unit, use of a sound-insulated enclosure, or low-noise solenoid designs. No significant geographical constraints are identified, confirming the adaptability of the system across different climates and locations.

Future work will focus on improving solenoid control using Programmable Logic Controllers (PLC) to expand frequency control and enhance system flexibility. The proposed pulsating flow technique will also be extended to other air-conditioning and refrigeration systems and evaluated with alternative refrigerants beyond ECO-22 to assess scalability and broader applicability.

## Empirical correlation results

The coefficient of performance (COP) is dependent on initial air temperature (*T*) and frequency (*f*). Based on the experimental measurements, the least squares method was used to calculate the empirical correlation coefficient. This fitted empirical correlation is utilized for predicting the COP across a wide range of initial air temperatures (*T*) and frequencies (*f*). Table 12 presents a comparison between the experimental measurements at (24, 26, and 28 °C) and (6, 9, and 12 Hz) compared with the empirical correlation response for COP.

**Table 12:** Comparison between experimental measurements and imperial correlation response for COP.

T_air, inlet_ (^o^C)	F (Hz)	Calculated COP	Imperial correlation response COP	Error (%)
24	9	1.26	1.251	0.71
26		1.27	1.236	2.68
28		1.23	1.221	0.73
26	6	1.23	1.220	0.81
	9	1.27	1.236	2.68
	12	1.20	1.208	0.71

**Fig. 13:**
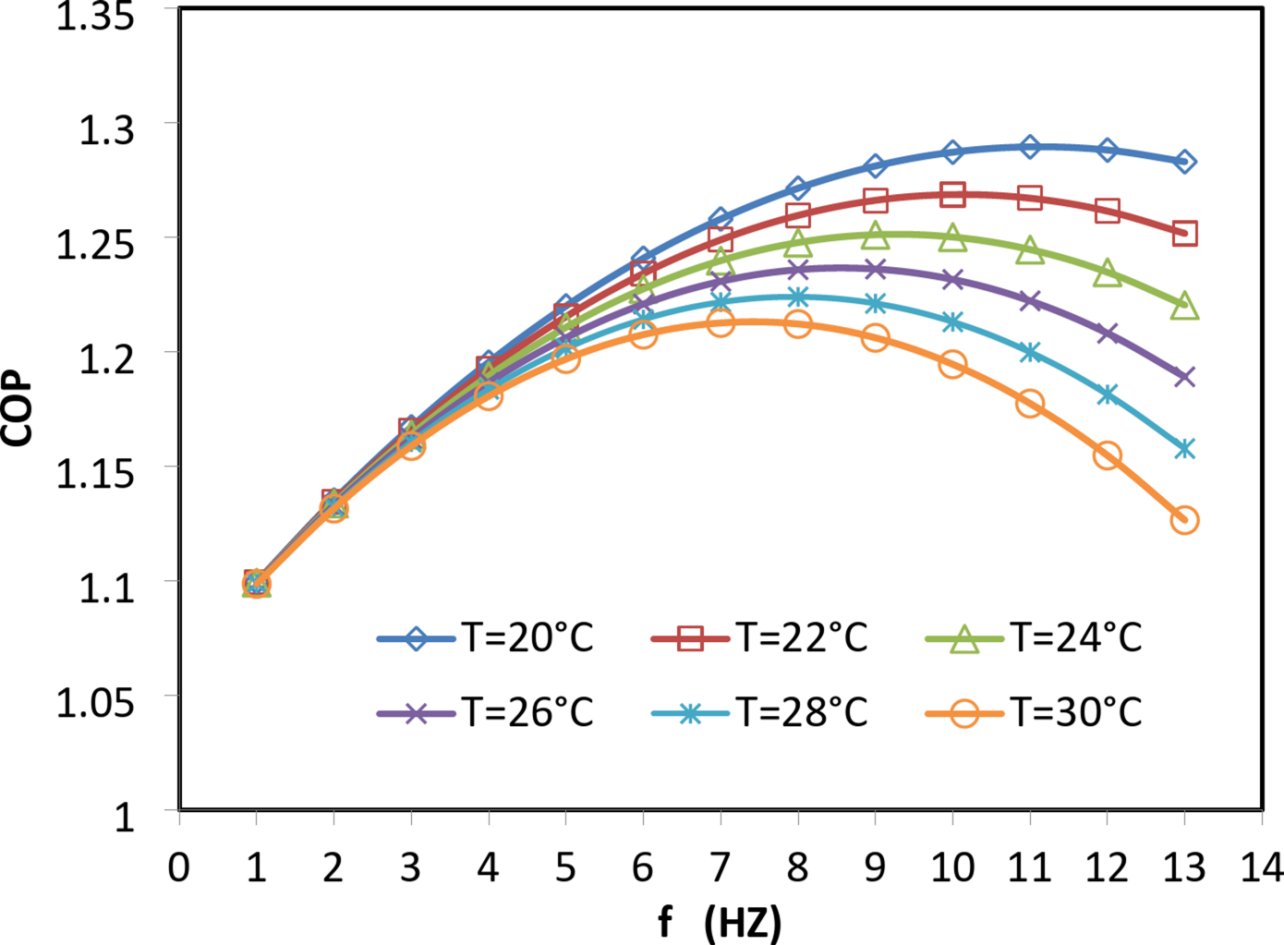
Predicted effects of pulsating refrigerant frequency and inlet air temperature on the COP.

Figure 13 illustrates the predicted effect of pulsating refrigerant of ECO-22 frequency and inlet air temperature on the COP. The COP is shown to increase with increasing pulsating air frequency and decrease with decreasing inlet air temperature. The effect of frequency on COP is particularly strong. An empirical correlation was developed to predict the COP at any inlet air temperature and frequency for a wide range, expressed by:

$$\:COP\hspace{0.17em}=\hspace{0.17em}a1f\:2\:T\hspace{0.17em}+\hspace{0.17em}a2f\:+\:a3$$

Where a least-squares fit gives a_1_ = − 9.26 × 10^− 5^, a_2_ = 0.04121, and a_3_ = 1.06018.

Therefore, the fitted equation is.

12 $${\rm COP =-9.26 \times 10^{-5} Tf^2 + 0.04121f + 1.06018}$$

## Conclusions

- Pulsating refrigerant flow generated by an electrical pulse control circuit at frequencies of 6, 9, and 12 Hz effectively enhanced the thermal performance of the evaporator and the overall system.
- Compared with continuous flow operation, the evaporator heat transfer rate increased by 13.33%, while the system coefficient of performance (COP) improved by 13.63%.
- The operating time required to reach the desired temperature was reduced by 23.3%, with the optimum performance achieved at a pulsation frequency of 9 Hz.
- The observed performance improvements were achieved with only a negligible increase in compressor power consumption, indicating enhanced efficiency without a significant energy penalty.
- The proposed modification is simple, cost-effective, and can be readily implemented in existing air-conditioning systems, demonstrating the potential of pulsating refrigerant flow for improving HVAC performance and reducing energy consumption.

## Future work

- Investigate the performance of the proposed pulsating flow air conditioning system under different ambient humidity levels and variable cooling loads to evaluate its applicability under a wider range of operating conditions.
- A detailed flow visualization and bubble pattern analysis are recommended in future studies to elucidate the heat transfer enhancement mechanisms associated with pulsating refrigerant flow.

## Data Availability

The data presented in this study are available on request from the corresponding author.
